# Impact of thyroid status and age on comprehensive geriatric assessment

**DOI:** 10.1007/s12020-013-0077-x

**Published:** 2013-11-01

**Authors:** Silvana Oliveira e Silva, I. Thien Chan, Maryna A. Lobo Santos, Marcela Cohen, Mayra de La Roque P. Araujo, Julia da Silva Almeida, Andressa Simões, Helder Renato B. Givigi, Mario Vaisman, Carlos M. Paixão, Patricia de Fatima dos S. Teixeira

**Affiliations:** 1 Faculdade de Medicina, Universidade Federal do Rio de Janeiro, Av. Carlos Chagas Filho, 373. Edifício do Centro de Ciências da Saúde, Bloco K, Cidade Universitária, Rio de Janeiro, RJ 21.941-902 Brazil; 2 Endocrinology Service, Hospital Universitário Clementino Fraga Filho, Rua Professor Rodolpho Paulo Rocco, 255 Cidade Universitária, Rio de Janeiro, RJ 21941-913 Brazil; 3 Universidade do Estado do Rio de Janeiro, Boulevard 28 de Setembro, 87, Vila Isabel, Rio de Janeiro, RJ 20551-030 Brazil

**Keywords:** Activities of daily living, Thyroid function, Subclinical hypothyroidism, Katz Index, HAQ

## Abstract

This study aimed to evaluate the prevalence of thyroid dysfunction in elderly subjects attending an outpatient clinic at a tertiary hospital and to assess whether subclinical hypothyroidism (SCH) or aging affected activities of daily living (ADLs), instrumental activities of daily living (IADLs), cognitive status, or depressive symptoms. This crosssectional study included 411 patients recruited in the outpatient geriatric setting. 48 subjects reported levothyroxine use and were evaluated separately. After excluding subjects with diseases or drugs which could influence thyroid status, the 284 subjects remaining were classified as having euthyroidism (*n* = 235, 82.8 %), subclinical hypothyroidism (*n* = 43, 15.1 %), subclinical hyperthyroidism (*n* = 4, 1.4 %), or overt hyperthyroidism (*n* = 2, 0.7 %). ADLs and IADLs were assessed using the Katz Index (ranging from 0 [independence] to 6 [dependence in all activities]) and Health Assessment Questionnaire (ranging from 0 to 3 [severely disabled]), respectively. Cognition was assessed using the mini mental state depressive symptoms that were assessed using the Geriatric depression scale or cornell scale for depression in dementia. SCH did not reduce performance in ADLs or IADLs in elderly subjects as a whole, but was an independent protective factor against dependence in ADLs (OR = 0.196 [0.045–0.853]; *p* = 0.003) and IADLs (OR = 0.060 [0.010–0.361]; *p* = 0.002) in subjects aged ≥85 years. Very old subjects with SCH showed better performance in ADLs than did those with euthyroidism (Katz Index: 0.9 ± 1.6 [median: 0.5] vs. 1.7 ± 1.7 [1.0], *p* = 0.024; HAQ: 1.2 ± 0.8 [0.9] vs. 1.8 ± 1.0 [1.9], *p* = 0.015). This putative protective effect of SCH was not found in subjects aged <85 years. The number of falls, number of medications used, depressive symptoms, and cognitive impairment did not differ among thyroid status groups, regardless of age. In conclusion, SCH does not have impact functional performance in the elderly population as a whole, but was associated with better functional status in subjects aged ≥85 years.

## Introduction

Thyroid hormones exert diverse effects on nervous system and overt thyroid dysfunctions are associated with several forms of cognitive impairments, peripheral neuropathies, and depressive or anxiety disorders [1]. Also, they act on muscular function [2], cardiovascular system [3, 4], erythropoiesis [5], and oxidative stress [6] among others organic functions. The implications of overt thyroid dysfunction in these systems may lead to high risk for morbid conditions and influence health related quality of life [7, 8] and mobility [9], which is an important marker of independence for activities of daily living (ALDs) in the old age.

Primary hypothyroidism is the most prevalent thyroid dysfunction in elderly individuals, and subclinical hypothyroidism, in which serum thyroid hormone levels are within normal range despite high serum thyroid-stimulating hormone (TSH) levels, is found in almost 20 % of the elderly population [10–15]. Recent researches have suggested that the upper reference value for serum TSH levels varies among age strata [16–19]. However, until now, there is no exact definition of these specific cutoffs, according to age, in our population [20–22].

High serum TSH levels have been associated with exceptional longevity, with significantly higher concentrations found in centenarians than in control subjects [23].

The progression rate from subclinical to overt hypothyroidism is associated with circulating antibodies against thyroid antigens, age, and baseline serum TSH levels [20]. Subclinical hypothyroidism was recently demonstrated to persist for 4 years in slightly more than half of subjects aged ≥65 years, with high rates of reversion to euthyroidism in individuals with minimally elevated serum TSH concentrations (<7.0 μUI/mL), who lacked circulating antibodies [24]. In the same study, a higher progression rate was not associated with subjects’ age or gender [24].

Endogenous subclinical hyperthyroidism is detected in 0.5–12.4 % of the general population, being more common in elderly women, especially in those living in iodine deficient areas [10–15]. The prevalence of low serum TSH in subjects taking levothyroxine is higher, reaching 30 % [13]. Whether endogenous and exogenous forms have the same clinical impacts on different populations remain unclear. Subclinical hyperthyroidism is associated with a higher risk of atrial fibrillation and reduced bone mass in elderly subjects [13, 25].

Large studies have failed to demonstrate an association between cognitive dysfunction and subclinical hypo or hyperthyroidism in elderly subjects [26, 27]. However, the results of the Invecchiare in Chianti Study [28] showed that abnormal cognitive function, detected by mini mental state examination (MMSE) scores less them 24 points, was more commonly associated with subclinical hyperthyroidism diagnosis [28]. In contrast, small studies applying very sensitive tests to evaluate specific cognitive domains have shown subtle changes in some cognitive areas (e.g., memory and executive function), but the clinical relevance of these results remains unknown [29–31].

Subclinical hypothyroidism was not found to be associated with dependence in ADLs, depressive symptoms, or cognitive function in a cohort of 599 subjects aged 85–89 years [32]. Furthermore, subjects from the same group, with mild elevations of serum TSH, had better walking speeds, and cardiopulmonary performance compared with those without thyroid dysfunction. Slightly elevated serum TSH levels were also associated with better global survive in another cohort, regardless of serum free triiodothyronine (FT3) levels, cognitive function, or global functional performance [32].

These findings have being considered in clinical practice to suggest that patients with minimal elevations of serum TSH should not be treated when their age are above a specific range [20–22, 33, 34]. A doubt is that regardless to possible improve quality of life of elderly subjects the restoration of euthyroidism might be associated with worse endpoints [20–22].

Recently, a differentiation in the approach of subclinical hypothyroidism in older people has been proposed, suggesting that L-T4 replacement should be avoid in the oldest old subjects, but should be considered in the modest old one, according to their cardiovascular risk [33]. If the impact of slightly elevations of serum TSH in ADLs, cognitive status, or depressive symptoms of elderly subjects will differ according to specific age grade, it is unknown yet.

The aim of the present study was to evaluate the prevalence of thyroid dysfunction in elderly subjects attending an outpatient clinic at a tertiary hospital. We also assessed whether thyroid function, according to different aging grade impacted instrumental activities of daily living (IADLs), ADLs, cognitive status, or depressive symptoms.

## Patients and methods

### Study design and population

Participants in this cross-sectional study were recruited from the outpatient geriatric clinic of Clementino Fraga Filho University Hospital, Federal University of Rio de Janeiro, between 2010 and 2012. All subjects ≥65 years old, who regularly attended the clinic were considered for inclusion. The institution’s ethics committee approved the study, and all patients provided written informed consent.

### Exclusion criteria

We excluded subjects with recent hospitalization (*n* = 39), recent radioiodine use (*n* = 16), and histories of amiodarone use (*n* = 21). Three individuals with serum free thyroxine (FT4) levels below the reference range, and inappropriately normal serum TSH levels were also excluded.

### Clinical evaluation

All included subjects were evaluated clinically via specific interviews, and all medical records were reviewed by one of the authors. The following data were collected: gender, age, anthropometric measures (body mass index, BMI obtained by direct or indirect measures) [34], recent hospitalizations, falls in the last year, comorbidities (diabetes mellitus, high blood pressure, history of coronary heart disease, heart failure [confirmed by echocardiographic evaluation], or cerebrovascular disease), history of thyroid disease or radioiodine use, tobacco use, and current medical prescriptions. Coronary heart disease was considered present when the patient had a past history of hospital admission for instable angina or myocardial infarct or even if the patient had a positive investigation for stable angina (invasive or noninvasive). Only past stroke history was considered as positive cerebrovascular disease present.

### Functional evaluation

Functional status was assessed using well-known scales adapted for and validated in Brazilian populations. ADLs (bathing, dressing, toileting, transferring, continence, and feeding) were assessed using the Katz Index [35–37]; a six-item interview which scores are ranging from 0 (independence in all six activities) to 6 (dependence in all activities). Any grade of dependence (scores ≥1) was considered as abnormal Katz Index. IADLs were assessed using the Brazilian version of the Health Assessment Questionnaire (HAQ) disability index [38–40]. The HAQ was originally designed to evaluate adults with arthritis, but it has been used to evaluate health in a wide range of research settings. It is based on a hierarchical model that considers disease effects in terms of death, disability, discomfort, side effects of treatment, and medical costs. Only the disability dimension, which comprises 20 questions about daily functioning during the past week, was used in the present study. These items are classified into eight components: dressing and grooming, arising, reaching, eating, gripping, walking, hygiene, and outdoor activities. Scores are ranging from 0 (completely self-sufficient) to 3 (severely disabled) [41]. Abnormal HAQ indexes were considered when the punctuations scored >1.

Cognition was assessed using the MMSE [42]. The cutoff value for cognitive impairment was modulated according to educational level for the Brazilian population, as suggested by Lourenço and Veras [43]. They proposed cutoff values of 24/30 for those with formal education (1 year at school and more) and 18/30 for those without.

Depressive symptoms were assessed using two instruments, according to cognition. For subjects with MMSE scores ≥13, we used the 15-item version of the Geriatric depression scale (GDS) [44, 45]. Scores ≥6/15 were considered to be suggestive of depressive disorder. For subjects with MMSE scores <13, the Cornell scale for depression in dementia (CSDD) was used [46, 47], with scores ≥10 indicating probable depressive disorder.

### Laboratory tests

Blood samples were collected at morning, and tested to determine TSH, FT4, and anti-thyroperoxidase antibody (TPO-Ab) levels using a third-generation chemiluminescence kit (Immulite 2000^®^; Diagnostic Products Corporation). The respective reference ranges were 0.4–4.0 μUI/mL, 0.8–1.9 ng/dL, and <35 UI/mL.

### Age and thyroid status classification

The study population was stratified into two age subgroups: old (<85 years) and very old (≥85 years). Subjects were also divided into five groups, according to thyroid status: (1) euthyroidism (TSH level within reference range), (2) subclinical hypothyroidism (TSH level >4.0–19.9 μUI/mL, FT4 level normal), (3) overt hypothyroidism (TSH level ≥20, FT4 level below reference range), (4) overt hyperthyroidism, (TSH level <0.4 μUI/mL, FT4 level above reference range), and (5) subclinical hyperthyroidism (TSH level <0.4 μUI/mL, FT4 level normal).

### Statistical analyses

All analyses were performed using SPSS software (ver. 13.0 for Windows; SPSS Inc., Chicago, IL, USA). Continuous variables were described as means ± standard deviations (medians) and compared between/among groups using Student’s *t* test or the Mann–Whitney test. Score punctuations for functional scales were assessed as continuous and categorical variables. The Kolmogorov–Smirnov test with Lilliefors correction was used to assess the Gaussian nature of pattern distributions. Proportions were compared using the Chi squared or Fisher’s exact test. Spearman correlation analyses were performed between serum TSH level and eight variables (age, BMI, number of falls in the past year, and Katz Index, HAQ, MMSE, GDS, and CSDD scores). Correlation values ≤0.20 were considered significant.

Binary logistic regression analysis was performed with abnormal IADL and ADL scores serving as dependent variables and confounding variables included in multivariate analysis (very old age [≥85 years], subclinical hypothyroidism, coronary artery disease, heart failure, cerebrovascular disease, depressive disorder, cognitive disorder, and ≥3 falls in the last year). All variables were included in each step model by entry method. A significance level of 5 % was used. Binary logistic regression analysis stratified by age group was also performed, including all previously mentioned variables, except very old age.

## Results

**Fig. 1:**
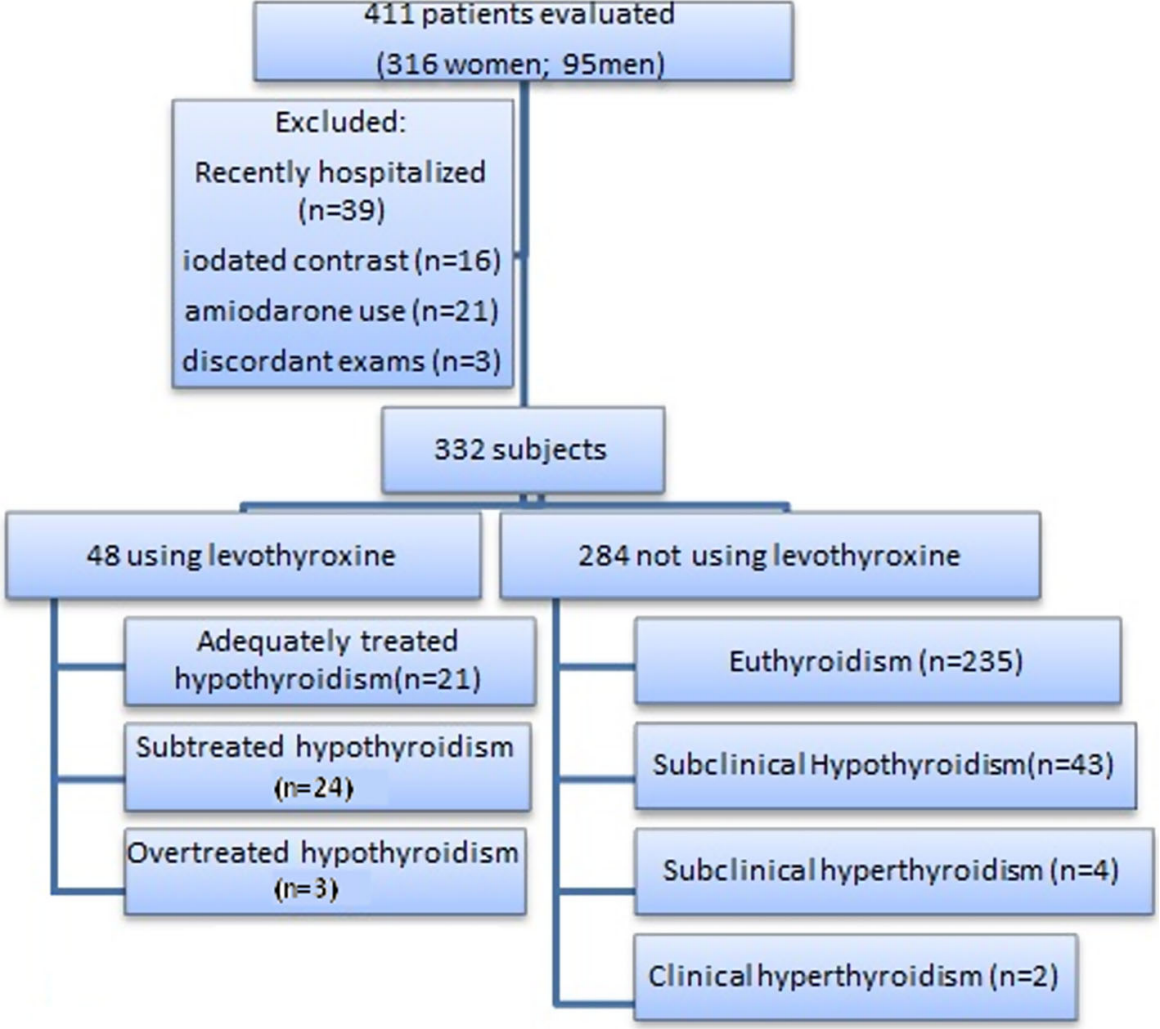
Population samples distribution in the study

Of 563 patients recruited in the outpatient geriatric setting, 411 (316 women and 95 men) provided informed consent and were included in the study. 48 subjects reported levothyroxine use and were evaluated separately. Exclusion criteria described previously were then applied (e.g., amiodarone or iodine contrast use and recent hospitalization). The remaining 284 individuals were categorized as having euthyroidism (*n* = 235, 82.8 %), subclinical hypothyroidism (*n* = 43, 15.1 %), subclinical hyperthyroidism (*n* = 4, 1.4 %), or overt hypothyroidism (*n* = 2, 0.7 %). Patients with overt hypothyroidism or subclinical hyperthyroidism were not submitted to further evaluation because of the small numbers of individuals in these groups. No case of overt hypothyroidism without hormone replacement was detected. The remaining 278 subjects had a mean age of 80.7 ± 67 years (range 65–102); 203 subjects were aged <85 years, and 75 were aged ≥85 years (Fig. 1).

### Patients using levothyroxine

After the initial application of exclusion criteria, we evaluated, apart from the whole group, 48 patients who were using levothyroxine at the time of evaluation (Fig. 1). They were divided into three subgroups: adequately treated (TSH within the reference range), undertreated (TSH >4.0 μUI/mL), and overtreated (TSH <0.4 μUI/mL) hypothyroidism. About half (*n* = 24) of these patients were adequately treated, 6.0 % (*n* = 3) were taking excessive levothyroxine doses, and 44.0 % (*n* = 21) were undertreated.

Undertreated subjects tended to have higher Katz Index scores (2.1 ± 1.9 [2.0]) than adequately treated (1.3 ± 1.3 [1.0]) and overtreated (1.7 ± 1.2 [1.0]) subjects, but this difference was not significant. Similarly, undertreated subjects tended to have higher HAQ scores than adequately and overtreated subjects (1.9 ± 0.9 [2.0] vs. 1.4 ± 0.8 [1.4] and 1.1 ± 1.2 [0.8], respectively; both *p* > 0.10). Adequately treated subjects’ performance in ADLs and IADLs was similar to that of subjects with euthyroidism and no levothyroxine replacement (Katz Index: 1.3 ± 1.3 [1.0] vs. 1.2 ± 1.6 [1.0], *p* = 0.21 and HAQ index: 1.4 ± 0.8 [1.4] vs. 1.4 ± 0.9 [1.4], *p* = 0.431). No significant difference among the three treated subgroups was found in any other variable (BMI, MMSE score, total number of medications in use, gender, TPO-Ab+, recurrent falls, the presence of cognitive deficit, depressive symptoms, cerebrovascular or coronary artery disease, and heart failure [data not shown]).

### Patients without levothyroxine replacement

General and functional characteristics of the study groups are shown in Table 1, according to thyroid status, including those without levothyroxine replacement. Demographic, functional, and risk-factor variables did not differ among subjects with euthyroidism and those with subclinical hypothyroidism.

**Table 1:** Group characteristics by thyroid status

	Subjects undergoing levothyroxine replacement (*n* = 48)			Subjects with no condition influencing thyroid function (*n* = 278)	
	Undertreated	Adequately treated	Overtreated	Euthyroidism	Subclinical hypothyroidism
*n* (%)	21 (44.0)	24 (50.0)	3 (6.0)	235 (84.5)	43 (15.4)
Age (years)	82.7 ± 6.8 (82.0)	79.9 ± 5.8(81.0)	81.3 ± 8.7 (79.0)	80.3 ± 6.8 (80.0)	81.2 ± 6.7 (83.0)
Women (%)	85.7	83.7	66.7	76.6	81.4
BMI (kg/m^2^)	33.9 ± 3.7 (26.6)	30.9 ± 5.8 (30.9)	27.5 ± 5.3 (26.4)	27.2 ± 4.7 (26.6)	27.0 ± 4.5 (27.7)
TSH (μUI/mL)	6.0 ± 3.0 (5.5)*	2,1 ± 1.2 (1.8)*	0.03 ± 0.05 (0.0)*	2.1 ± 0.9 (2.0)*	5.5 ± 1.2 (5.1)*
FT4 (ng/dL)	1.1 ± 0.2 (1.1)	1.2 ± 0.2 (1.2)^#^	1.6 ± 0.3 (1.5)^#^	1.1 ± 0.2 (1.1)	1.1 ± 0.14 (1.0)
TPO-Ab+ (%)	53.0	52.0	0.0	12.2	10.5
Medications in use (*n*)	8.2 ± 2.7 (7.0)	8.5 ± 2.6 (8.0)	7.9 ± 2.7 (7.0)	6.4 ± 2.8	6.8 ± 2.8
≥3 falls in the last year	14.3	4.2	0	11.6	9.3
Katz Index	2.1 ± 1.9 (2.0)	1.3 ± 1.3 (1.0)	1.7 ± 1.2 (1.0)	1.2 ± 1.6 (1.0)	1.2 ± 1.7 (1.0)
Functional dependence-Katz Index (%)	81.0	78.3	100	57.4	60.5
HAQ	1.9 ± 0.9 (2.0)	1.4 ± 0.8 (1.4)	1.1 ± 1.2 (0.8)	1.4 ± 0.9 (1.4)	1.4 ± 0.9 (1.4)
Functional dependence- HAQ (%)	71.4	69.6	33.3	59.1	55.8
MMSE	19.7 ± 7.3(22.0)	22.1 ± 7.6 (24.0)	20.0 ± 7.2 (21.5)	19.3 ± 8.2 (20.0)	19.7 ± 7.9 (20.0)
Cognitive deficit (%)	57.1	45.8	33.3	54.1	60.5
Depressive disorder (%)	57.1	54.2	0.0	45.6	52.4
CAD (%)	5.6	8.3	33.3	9.4	2.4
Heart failure (%)	22.2	16.0	0.0	32.8	35.7
CVD (%)	25	21.7	27.8	15.9	21.4

Values are presented as *n* (%) or mean ± standard deviation (median)

*SD* standard deviation, *BMI* body mass index, *TSH* thyroid-stimulating hormone, *FT4* free thyroxine, *TPO-AB* anti-thyroperoxidase antibody, *HAQ* health assessment questionnaire, *CAD* coronary artery disease, *CVD* cerebrovascular disease

* *p* < 0.001 comparing subjects with euthyroidism and SHypo, and also comparing three treat groups; ^#^ *p* < 0.001 comparing overtreated and adequately treated subjects; No other significant difference was observed between subjects with SHypo and euthyroidism, among treated groups, or between those with euthyroidism and adequately treated subjects

ADL and IADL scores are shown in Table 2 according to age group, independent of thyroid status. As expected, ADL and IADL scores were significantly higher among very old subjects than among old individuals (Katz Index: 1.6 ± 1.7 [1.0] vs. 1.1 ± 1.6 [1.0], *p* = 0.001; HAQ: 1.7 ± 0.8 [1.6] vs. 1.3 ± 0.9 [1.1], *p* < 0.001) and very old subjects had more cognitive impairment (71.6 vs. 49.0 %, *p* = 0.001).

**Table 2:** Subjects characteristics by age group

Variable	<85 years	≥85 years
*n* (%)	203 (73)	75 (27)
Age (years)	77.4 ± 4.8 (78)*	88.6 ± 3.7 (88.0)*
Women (%)	76.8	78.7
BMI (kg/m^2^)	27.6 ± (27.2)^†^	26.1 ± (26.2)^†^
TSH (μUI/mL)	2.5 ± 1.5 (2.1)	2.9 ± 1.7 (2.6)
FT4 (μUI/mL)	1.1 ± 0.1 (1.1)	1.2 ± 0.2 (1.1)
TPO-Ab+ (%)	11.4	13.3
Falls in last year	1.2 ± 1.9 (1.0)	0.9 ± 1.3 (0.0)
Medications used	6.6 ± 2.7 (7.0)	6.2 ± 2.8 (6.0)
Katz Index	1.1 ± 1.6 (1.0)**	1.6 ± 1.7 (1.0)**
Functional dependence, Katz Index (%)	51.7**	74.7**
HAQ	1.3 ± 0.9 (1.1)*	1.7 ± 0.8 (1.6)*
Functional dependence, HAQ (%)	52.2***	76.0***
Cognitive deficit (%)	49.0**	71.6**
Depressive symptoms (%)	44.2	53.5
CVD (%)	17.0	16.2
Heart failure (%)	31.0	39.2
CAD (%)	7.5	10.8

Continuous variables are presented as mean ± standard deviation (median)

*BMI* body mass index, *TSH* thyroid-stimulating hormone, *FT4* free thyroxine, *TPO-Ab* anti-thyroperoxidase antibody, *HAQ* health assessment questionnaire, *CVD* cerebrovascular disease, *CAD* coronary artery disease

* *p* = 0.000; ** *p* = 0.001; *** *p* < 0.001; ^†^ *p* = 0.023

**Fig. 2:**
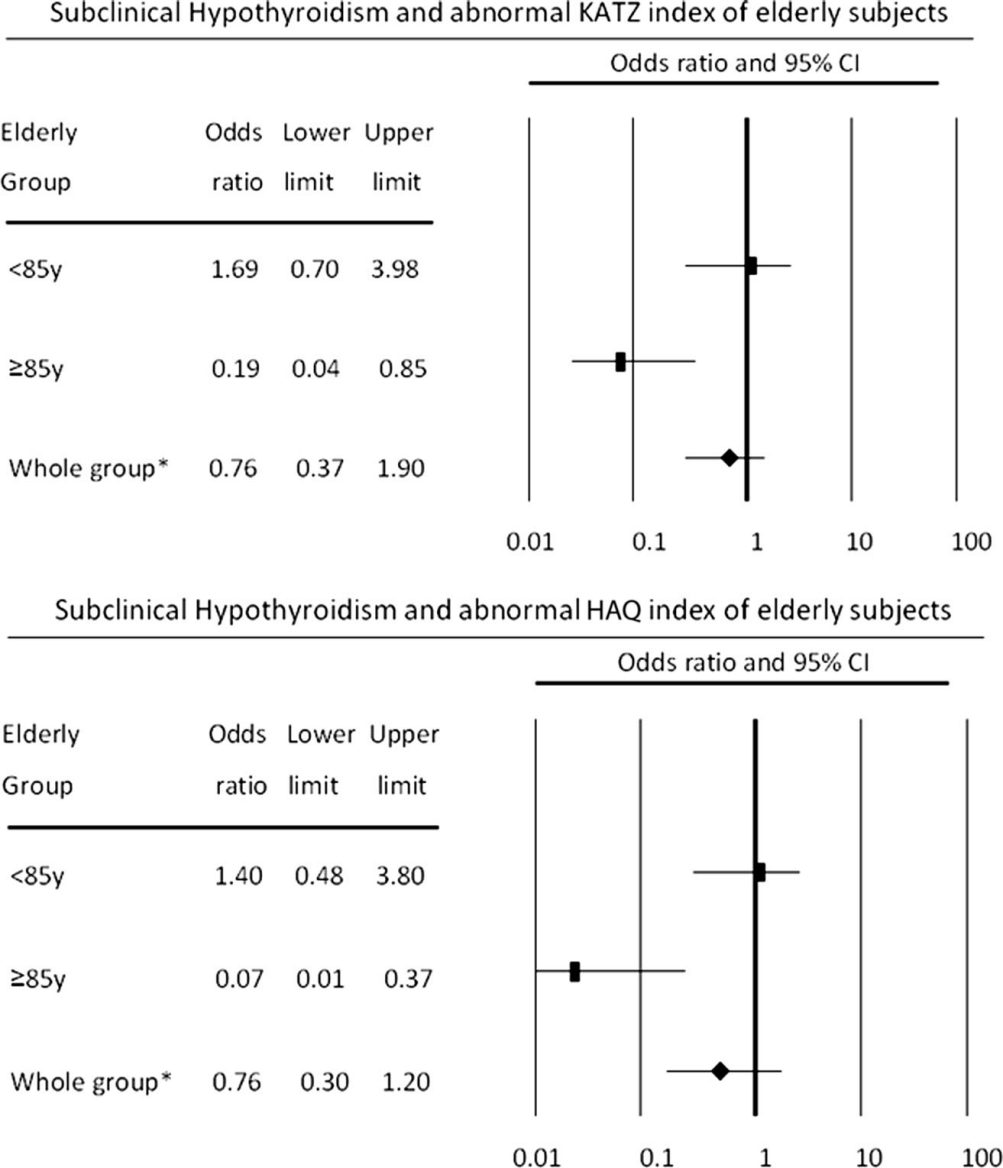
Adjusted for the presence of cognitive defect, depressive disorder, coronary artery disease, cerebrovascular disease, heart failure, and recurrent falls. *asterisk* indicates additional adjust for very old age

Results of multivariate analyses are shown in Tables 3 and 4. In the group as a whole, the risk of dependence in ADLs, as measured by the Katz Index, was independently related to cognitive impairment (odds ratio, OR = 2.555, 95 % confidence interval, CI 1.506–4.334; *p* = 0.001) and very old age (OR = 2.381, 95 % CI 1.249–4.538; *p* = 0.008), but was not related to the presence of subclinical hypothyroidism (OR = 0.765, 95 % CI 0.369–1.899). The risk of abnormal HAQ score was independently related to very old age (OR = 2.816, 95 % CI 1.451–45.468; *p* = 0.002), depressive disorder (OR = 1.830, 95 % CI 1.063–3.150; *p* = 0.029), and cognitive impairment (OR = 2.422, 95 % CI 1.413–4.154; *p* = 0.001). Evaluating the whole group, subclinical hypothyroidism did not reduce elderly subjects’ performance in ADLs or IADLs (Fig. 2).

**Table 3:** Variables independently associated with a high risk for functional dependence, measured by the Katz Index

	Beta coefficient	Standard error	*p*	OR	95 % CI	
					Lower	Upper
Whole group						
Cognitive deficit^a^	0.938	0.270	0.001	2.555	1.506	4.334
Very old age^b^	0.867	0.329	0.008	2.381	1.249	4.538
Old (<85 years)						
Cognitive deficit^b^	1.005	0.311	0.001	2.733	1.486	5.029
Very old (≥85 years)						
Subclinical hypothyroidism^b^	−1.630	0.750	0.030	0.196	0.045	0.853

*OR* odds ratio, *CI* confidence interval

^a^ Binary logistic regression model adjusted for depressive disorder, cognitive deficit, the presence of subclinical hypothyroidism, coronary artery disease, heart failure, recurrent falls, very old age, and cerebrovascular disease

^b^ Binary logistic regression model adjust for all factors listed above except very old age

**Table 4:** Variables independently associated with a high risk for functional dependence, measured by the HAQ

	Beta coefficient	Standard error	*p*	OR	95 % CI	
					Lower	Upper
Whole group^a^						
Cognitive Impairment	0.885	0.329	0.001	2.422	1.413	4.154
Very old age	1.035	0.338	0.002	2.816	1.451	5.468
Depressive disorder	0.604	0.456	0.029	1.830	1.063	3.150
Old (<85 years)^b^						
Cognitive deficit	1.024	0.314	0.001	2.785	1.504	5.158
Depressive disorder	0.836	0.319	0.009	2.307	1.235	4.308
Very old (≥85 years)^b^						
Subclinical hypothyroidism	−2.813	0.916	0.002	0.060	0.010	0.361

*OR* odds ratio, *CI* confidence interval

^a^ Binary logistic regression model adjusted for depressive disorder, cognitive deficit, the presence of subclinical hypothyroidism, coronary artery disease, heart failure, recurrent falls, very old age, and cerebrovascular disease

^b^ Binary logistic regression model adjust for all factors listed above except very old age

#### Impact of thyroid status on functional status of patients without levothyroxine replacement, stratified by age

**Fig. 3:**
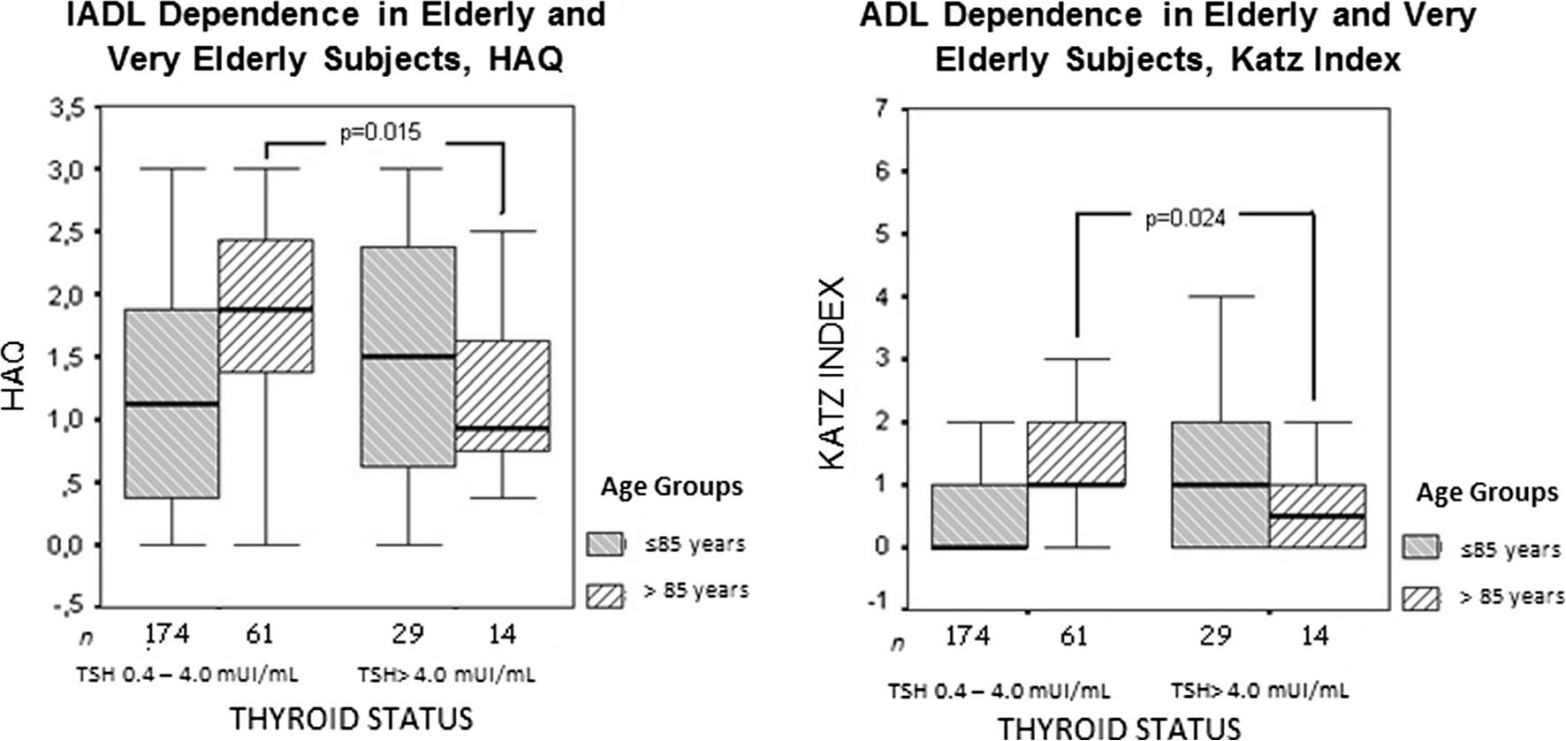
Comparisons between HAQ e Katz Indexes from elderly patients with SHypo versus “normal” serum TSH according to different age subgroups

ADL scores were lower in very old subjects with subclinical hypothyroidism than in those with euthyroidism (Katz Index: 0.9 ± 1.6 [0.5] vs. 1.7 ± 1.7 [1.0], *p* = 0.024; HAQ: 1.2 ± 0.8 [0.9] vs. 1.8 ± 0.8[1.9], *p* = 0.015; Table 5). This putative protective effect of subclinical hypothyroidism was not found in subjects aged <85 years (Fig. 3). The number of falls, number of medications used, depressive symptoms, and cognitive impairment did not differ among thyroid status groups, regardless of age.

**Table 5:** Functional profiles in subjects with euthyroidism and subclinical hypothyroidism, by age group

Variable	<85 years		≥85 years	
Thyroid status	Euthyroidism	Subclinical hypothyroidism	Euthyroidism	Subclinical hypothyroidism
*n*	174	29	61	14
Katz Index	1.00 ± 1.5 (0.0)	1.4 ± 1.7 (1.0)	1.7 ± 1.7 (1.0)*	0.9 ± 1.6 (0.5)*
Functional dependence, Katz Index (%)	49.4	65.5	80.3^†^	50.0^†^
HAQ	1.2 ± 0.9 (1.1)	1.5 ± 1.0 (1.5)	1.8 ± 0.8 (1.9)**	1.2 ± 0.8 (0.9)**
Functional dependence, HAQ (%)	44.8	62.1	78.7^α^	35.7^α^
Recurrent falls (%)^a^	11.0	10.3	13.6	7.1
Medications used	6.5 ± 2.7 (6.0)	7.4 ± 2.6 (7.0)	6.4 ± 2.9 (6.0)	5.4 ± 2.6 (5.5)
Cognitive deficit (%)	48.6	51.7	70.0	78.6
Heart failure	29.7	39.3	41.7	29.0
Coronary artery disease	8.1	3.6	13.3	0.0
Cerebrovascular disease	15.1	28.6	18.3	7.1
Depressive disorder (%)	44.4	42.9	49.1	71.4

Continuous variables are presented as mean ± standard deviation (median)

*SHypo* subclinical hypothyroidism, *HAQ* health assessment questionnaire

* *p* = 0.024; ** *p* = 0.015; ^†^ *p* = 0.036; ^α^ *p* = 0.004

^a^ Three or more falls in the last year

Age-stratified analysis revealed a weak positive correlation between serum TSH level and MMSE score in patients aged <85 years (*rs* = 0.144, *p* = 0.021), but no correlation between TSH level and any other variable in this subgroup (data not shown). In the very old group, no correlation was found between serum TSH level and any other studied variable (data not shown).

In patients aged <85 years, only cognitive deficit was an independent risk factor for dependence in ADLs, assessed by the Katz Index (OR = 2.733, 95 % CI 1.486–5.029; *p* = 0.001). The risk of dependence in IADLs, assessed by the HAQ, was independently related to cognitive deficit and the presence of a depressive disorder (Tables 3, 4). Subclinical hypothyroidism did not influence the risk of dependence in ADLs or IADLs (Tables 3, 4; Fig. 2). In the very old group, subclinical hypothyroidism was an independent protective factor related to dependence in ADLs (OR = 0.196, 95 % CI 0.045–0.853; *p* = 0.003) and IADLs (OR = 0.060, 95 % CI 0.010–0.361; *p* = 0.002; Tables 3, 4; Fig. 2).

## Discussion

Our study findings indicate that thyroid status has no impact on functional performance in the elderly population as a whole, but patients aged <85 years cannot be evaluated in the same manner as very old subjects. Measurement of the impact of thyroid function in elderly individuals using different endpoints according to age has recently been proposed [15, 20–22, 33, 48, 49]. The results of the present study showed the protective effect of subclinical hypothyroidism on performance in ADLs and IADLs in very elderly people. Although subjects aged ≥85 years had more comorbidities and worse functional performance than those aged <85 years, these very old subjects when presented with serum TSH levels above the reference range showed better performance in ADLs and IADLs than euthyroid very old patients in same age group. These results are in accordance with previously published data on mobility in elderly individuals [9, 32]. Several studies have presented evidence supporting a change in the distribution of the serum TSH normality curve according to age, with a shift to higher values for very old individuals [16, 17, 49, 50]. This change, underlain by genetic factors, modifications in central set-point for thyroid hormone feed-back, and dietary iodine supply, would provide better guidance for further evaluation of thyroid dysfunction and would reflect growing support for the assumption that minimally elevated serum TSH levels in elderly subjects should be considered normal [16, 17, 49, 50]. Very old individuals with high thyrotropin levels do not experience adverse effects or have higher mortality rates, and thus may not require levothyroxine replacement [21–23, 32].

Few large studies have examined associations between functionality and thyroid function [9, 32]. More such studies are necessary, given the evidence for an association between a subtle reduction in thyroid activity and better functionality in elderly individuals.

Comorbidities were prevalent, and the risks of frailty and functional decline were high in the population of the present study, which was recruited in an outpatient setting. Few studies have examined thyroid status in similar populations due to the difficulties presented by the prevalence of adverse endpoints and the high risk of follow-up interruption. Thus, the results of the present study should be compared with data from populations with similar characteristics.

As expected, dependence in ADLs and IADLs and cognitive decline were more prevalent in very old than in old subjects, reflecting a global decline related to aging [50–52]. We also found lower BMIs in very old people than in the old group. Both age strata are eutrophic, but these lower values may be associated with changes in body composition, muscle loss, and frailty in very old subjects; additional research is needed to define the associations between body composition and thyroid function in old and very old subjects. Despite these results, functional performance differed according to serum TSH level in very old subjects, as reported above.

The prevalence of thyroid disease in this study was similar to that reported in previous studies, although the prevalence of subclinical hypothyroidism was almost three-fold higher than that found in a study of community-based elderly Brazilians [18].

Since almost 14.5 % of our study population used levothyroxine, and no subject had been diagnosed with thyroid cancer, we considered these patients to be under treatment for primary hypothyroidism. We observed a high prevalence of undertreated patients, but some authors have shown that about 60 % of patients receiving levothyroxine have euthyroidism [13], similar to the results of the present study.

Subjects undergoing levothyroxine replacement with elevated serum TSH levels tended to show worse performance in ADLs than those adequately treated, although this difference was not significant. These data must be interpreted with caution, given the small sample size. These endpoints appeared to be similar in patients with euthyroidism and adequately treated subjects.

We found no significant association between cognitive and thyroid functions in the present study. Memory alterations and reduced cognitive performance are the most common issues in patients with OH [1]. Some trials have shown improved cognitive function after levothyroxine replacement, but results have not been uniform, and no age-stratified analysis has demonstrated such an effect [26, 27, 29–31]. Studies of correlations among age, subclinical hypothyroidism, and cognition are difficult to perform because cognitive function declines with age. Most longitudinal studies have failed to demonstrate that elevated serum TSH is related to cognitive effects, which may be related to methodological issues [26]. MMSE, the screening tool for cognitive decline used in this study, is not a very sensitive test, besides it is largely employed in the literature despite this limitation. Additional studies using more specific methodologies to examine cognitive function in elderly patients with subclinical hypothyroidism, and the practical repercussions of any such association are needed. Given the relationship between depression and cognition, some studies have demonstrated an association between minimal elevations on serum TSH and depressive symptoms in elderly individuals [53, 54], but other studies using several screening tools have found no such association [55, 56]. In the present study, no difference in depressive symptoms was observed between patients with euthyroidism and hypothyroidism, irrespective to age.

Limitations of this study include the characteristics of the population, which was highly selected in an outpatient setting, resulting in a high prevalence of comorbidities, use of multiple medications, and low functional status. Population-based studies are needed to corroborate our findings. Some losses of study subjects occurred and were related to their dependence on proxies or caregivers to escort them to the clinic for complete evaluation. This last limitation is also associated with the small number and the characteristics of a convenient selected sample, which might influence some negative results in the study. The well-known limitations of cross-sectional studies’ results also apply to the present study. Prospective studies should be conducted to more thoroughly explore our findings. Few studies have assessed thyroid status in elderly, especially very elderly, individuals, who represent the most rapidly growing age group worldwide. Better knowledge of this and other aspects in the very elderly population is urgently needed.

## Conclusions

Subclinical hypothyroidism does not impact functional performance in the elderly population as a whole. However, serum TSH above the reference range may be associated with better functional status in subjects aged ≥85 years. More thorough studies are needed to assess these findings and their impact on hospitalization and mortality in this specific population.
